# Integrated Weighted Gene Co-expression Network Analysis Identified That *TLR2* and *CD14* Are Related to Coronary Artery Disease

**DOI:** 10.3389/fgene.2020.613744

**Published:** 2021-01-26

**Authors:** Bin Qi, Jian-Hong Chen, Lin Tao, Chuan-Meng Zhu, Yong Wang, Guo-Xiong Deng, Liu Miao

**Affiliations:** ^1^Departments of Cardiology, Liuzhou People's Hospital, Liuzhou, China; ^2^Departments of Cardiology, The First People's Hospital of Nanning, Nanning, China

**Keywords:** gene expression omnibus, integrated weighted gene co-expression network analysis, functional enrichment, functional validation, prognostic analysis, coronary artery disease

## Abstract

The current research attempted to identify possible hub genes and pathways of coronary artery disease (CAD) and to detect the possible mechanisms. Array data from GSE90074 were downloaded from the Gene Expression Omnibus (GEO) database. Integrated weighted gene co-expression network analysis (WGCNA) was performed to analyze the gene module and clinical characteristics. Gene Ontology annotation (GO), Disease Ontology (DO) and the Kyoto Encyclopedia of Genes and Genomes (KEGG) pathway enrichment analyses were performed by clusterProfiler and the DOSE package in R. A protein-protein interaction (PPI) network was established using Cytoscape software, and significant modules were analyzed using Molecular Complex Detection (MCODE) to identify hub genes. Then, further functional validation of hub genes in other microarrays and population samples was performed, and survival analysis was performed to investigate the prognosis. A total of 660 genes were located in three modules and associated with CAD. GO functions identified 484 biological processes, 39 cellular components, and 22 molecular functions with an adjusted *P* < 0.05. In total, 38 pathways were enriched in KEGG pathway analysis, and 147 DO items were identified with an adjusted *P* < 0.05 (false discovery rate, FDR set at < 0.05). There was a total of four modules with a score > 10 after PPI network analysis using the MCODE app, and two hub genes (*TLR2* and *CD14*) were identified. Then, we validated the information from the GSE60993 dataset using the GSE59867 dataset and population samples, and we found that these two genes were associated with plaque vulnerability. These two genes varied at different time points after myocardial infarction, and both of them had the lowest prognosis of heart failure when they were expressed at low levels. We performed an integrated WGCNA and validated that *TLR2* and *CD14* were closely associated with the severity of coronary artery disease, plaque instability and the prognosis of heart failure after myocardial infarction.

## Introduction

With the continuous improvement of living standards, the incidence of coronary artery disease (CAD) is also increasing. According to the latest epidemiological studies, the number of CAD patients will increase rapidly in the next 10 years (Chen et al., 2018). As a multifactorial and complex disorder, many genetic complications and environmental exposures, including early family history, unhealthy life habits (such as smoking and excessive drinking), hypertension, dyslipidemia, obesity and diabetes, can result in CAD (Yamada et al., 2015; Abram et al., 2017; Chiu et al., 2018; Madhavan et al., 2018). At the same time, improvements in science and technology have provided us with a new understanding of CAD that abnormal gene expression plays an important role in the pathogenesis of CAD (Malakar et al., 2019).

With the remarkable evolution of bioinformatics, numerous microarray data can be used to identify hub genes, interaction networks and pathways of CAD. However, detection of these characteristics remains challenging. Weighted gene co-expression network analysis (WGCNA) (Miao et al., 2019a) is a highly used systems biology method that enables integrated analysis of gene expression and clinical traits. We constructed an mRNA expression profile of CAD samples to screen gene modules that are closely related and highly coregulated with CAD to show the potential molecular mechanisms.

Here, our current study analyzed CAD gene expression profile datasets from the Gene Expression Omnibus (GEO) database by constructing an integrated WGCNA strategy to consult the molecular mechanisms of CAD and to identify hub genes as theoretical prognostic molecules. Then, we further validated the selected hub genes in another dataset to determine whether hub genes play a role in plaque stability. Finally, we validated the relationship between the expression of hub genes and heart failure after myocardial infarction.

## Materials and Methods

### Gene Expression Profile and Probe Reannotation

A total of three datasets were downloaded from GEO (https://www.ncbi.nlm.nih.gov/geo/). GSE90074 (Ravi et al., 2017) was based on the platform of GPL6480 Agilent-014850 Whole Human Genome Microarray 4x44K G4112F and contained 143 samples (93 obstructive CAD samples and 50 controls). We used a numerical scale (from 0 to 4) to identify the degree of stenosis in the coronary arteries, with higher numbers indicating more severe stenosis. A coronary artery is defined as 0 when all major coronary arteries have <10% stenosis, 1 when any one coronary artery except the left main coronary artery has 10–70% stenosis, 2 when only one coronary artery has more than 70% stenosis, 3 when two coronary arteries have more than 70% stenosis, 3 when three vessels have more than 70% stenosis and 4 when the left main has more than 50%. GSE60993 (Park et al., 2015) was based on the platform of the Illumina HumanWG-6 v3.0 expression bead chip. This dataset included nine unstable angina (UA) samples, 10 non-ST-segment elevation acute myocardial infarction (NSTEMI) samples, seven ST-segment elevation acute myocardial infarction (STEMI) samples and seven normal controls. We used the GSE60993 dataset to verify the relationship between changes in hub gene expression and plaque vulnerability. GSE59867 (Maciejak et al., 2015) was based on the platform of GPL6244 Affymetrix Human Gene 1.0 ST Array and collected peripheral blood samples from patients (*n* = 111) with STEMI at four time points (admission, discharge, 1 month after MI, and 6 months after MI). We used the GSE59867 dataset to verify the relationship between the changes in hub gene expression and the incidence of heart failure after myocardial infarction. The CEL file in the microarray was annotated into an expression value matrix by using the Affy package in R, and then, the probe information in the microarray was annotated into gene names. This step was done using the Bioconductor package (hgu133plus2, illuminaHumanv3 and hugene10sttranscriptcluster) in R software (Miao et al., 2018). If the expression value of multiple probe information corresponded to a gene with the same name, the average value was selected as the expression level of that gene. The flowchart can be found in Supplementary Figure 1.

### Weighted Gene Co-expression Network Analysis and Module Preservation

WGCNA, which constructs a scale-free network by correlating gene expression levels with clinical features, is often used for a variety of systematic biological analyses. To ensure that the results of network construction were reliable, we normalized the samples first and then removed the outlier samples. The soft threshold power must be selected according to the standard scale-free networks, and all differential genes were calculated by a power function. Subsequently, the adjacency matrix was transformed into a topological overlap matrix (TOM), and the corresponding dissimilarity (1-TOM) was calculated. The dynamic tree cut method was performed to identify the module by hierarchically clustering genes. A deepSplit value of 2 and a minimum size cutoff of 30 were selected as the distance measure for the resulting dendrogram. A height cutoff of 0.25 was used as the standard to merge highly similar modules. Then, we used the WGCNA package (Langfelder and Horvath, 2008) to run the module preservation function.

### Finding Module of Interest

We performed Pearson's correlation tests to assess the correlation between clinical characteristics and modules and to identify the meaningful modules. Subsequently, we defined the correlation of the gene expression profile with module eigengenes (Mes) as a module membership (MM), and the correlation (the absolute value) between outer features and gene expression profiles were defined as the gene significance (GS). Then, we performed further analyses for the genes located in the modules of interest with the highest MM and highest GS values.

### Functional Annotation and Hub Genes

Gene Ontology (GO), Kyoto Encyclopedia of Genes and Genomes (KEGG) pathway enrichment and Disease Ontology (DO) analyses are three very important components, so we employed clusterProfiler and the DOSE package in R (Yu et al., 2012) to analyze all genes in the modules of interest and to determine the possible mechanisms by which module genes play a role in the correlative clinical features. The cutoff criteria were set at *P*-value < 0.05 and false discovery rate (FDR) < 0.1. Cytoscape software was employed to visualize and construct a protein-protein interaction (PPI) network, and molecular complex detection (MCODE) (Bader and Hogue, 2003; Shannon et al., 2003) was used to analyze the most notable clustering module. An MCODE score > 8 was a threshold for the next analysis. Subsequently, we selected two genes with the highest semantic similarity value for verification in the GoSemSim package (Yu et al., 2010) in R.

### Hub Gene Validation and Survival Analysis

First, to further understand the relationship between hub genes and plaque vulnerability, we downloaded GSE60993 and used it as the training dataset. Then, we compared the expression differences of hub genes among different groups and showed the results in the ggplot2 package (Miao et al., 2019b) in R. Subsequently, the “survival” package (Zhu et al., 2019) in R was used to perform overall survival (heart failure) and disease-free survival analyses for all hub genes. Patients were divided into four groups (high vs. low) based on the hub gene expression level in comparison to the mean expression level of that hub gene. Furthermore, dataset GSE59867, which includes 111 acute myocardial infarction (AMI) samples in which survival time was provided, was used to test the significance of hub genes for heart failure survival. A Kaplan-Meier survival plot was also constructed.

In total, we collected clinical information from 1,029 patients who presented to Liuzhou People's Hospital from June 1, 2018, to May 5, 2019, with acute chest pain and were admitted for coronary angiography. CAD, UA and AMI were diagnosed based on the Fourth Universal Definition of Myocardial Infarction (2018) (Zhuo et al., 2019). Exclusion criteria included subjects with contrast agent sensitivity, incomplete clinical data, poor compliance, obvious surgical contraindications and autoimmune diseases. Biochemical measurements, diagnostic criteria and collected clinical data can be found in our previous manuscript (Miao et al., 2017). Consent was obtained from all subjects for this study, and the Ethics Committee of Liuzhou People's Hospital approved this study (No: Lunshen-2018-KY; Jan. 06, 2018). The study adhered to the Declaration of Helsinki of 1975 (http://www.wma.net/en/30publications/10policies/b3/) and its revision in 2008.

The procedures of blood sample collection, RNA isolation, reverse transcription cDNA and RT-qPCR were the same as those in our previous studies and were carried out in strict accordance with the product instructions and laboratory operating procedures (Miao et al., 2019c). Specific divergent primers were designed to amplify the transcripts and are shown in Supplementary Table 1. The statistical software packages SPSS 22.0 (SPSS Inc., Chicago, IL, USA) and R software (version 3.6.0) were used for statistical analyses. According to the statistical specification, quantitative variables were presented according to the means ± standard deviations, and since TG did not conform to a normal distribution, we used the Wilcoxon-Mann-Whitney test for analysis. Comparisons of percentages between groups were performed by means of the chi-square test. All *P*-values were two-sided and considered statistically significant according to *P* < 0.05.

## Results

### Data Preprocessing

When analyzing GSE90074, we first judged the sample quality. After controlling for quality, all of the samples were well normalized (Supplementary Figure 2). After removing duplicate values and annotating probes, a total of 54,560 expression probes obtained in our expression microarray yielded a matrix of 19,537 gene expression values.

### Weighted Gene Co-expression Networks

**Figure 1:**
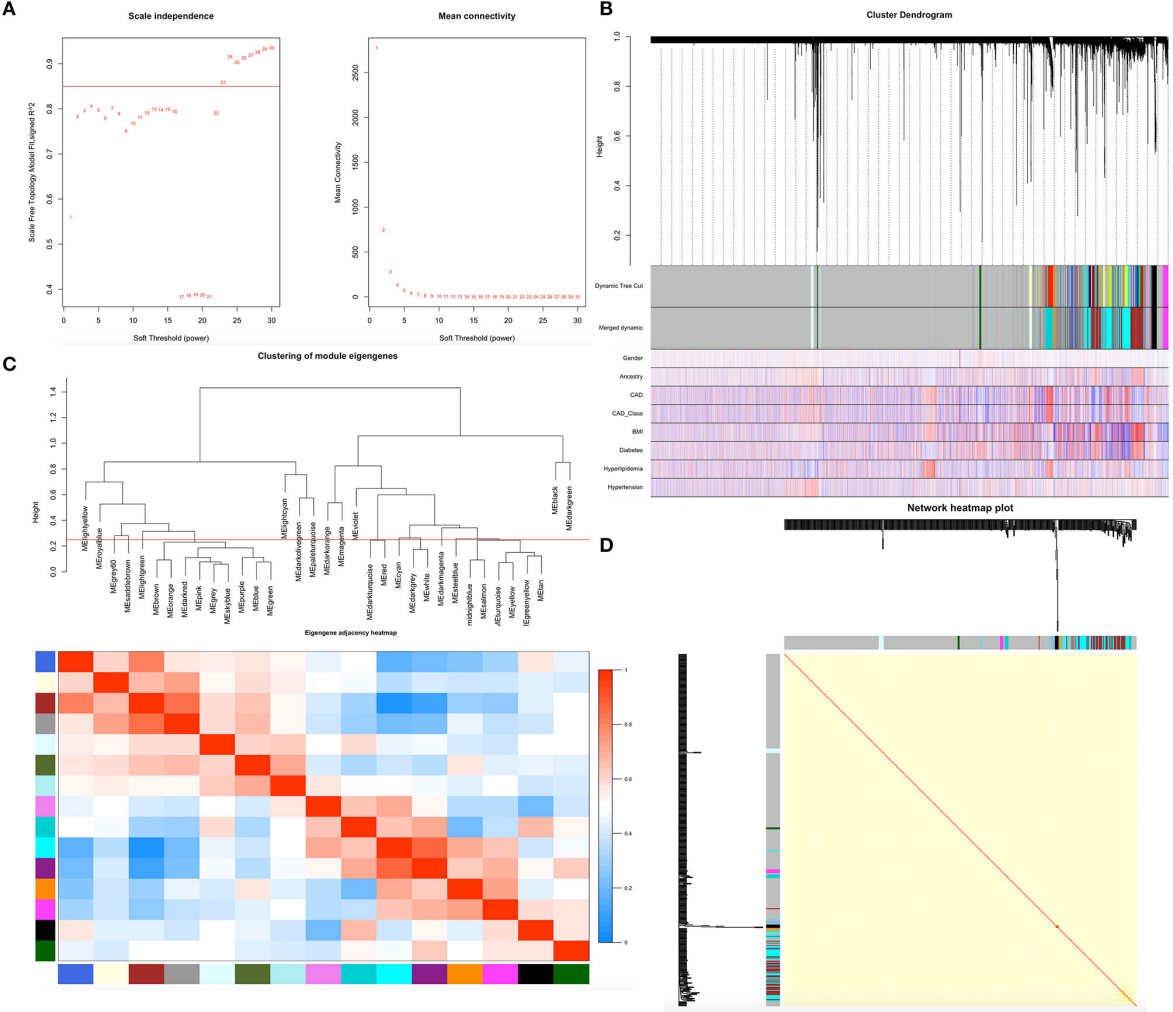
Demonstration of the WGCNA process. **(A)** Analysis of network topology for various soft-thresholding powers. **(B)** Clustering dendrogram of genes. Gene clustering tree (dendrogram) obtained by hierarchical clustering of adjacency-based dissimilarity. **(C)** Relationship among all the modules. **(D)** Heatmap of the topological overlap in the gene network.

Before constructing the weighted co-expression network, we chose the soft-threshold β parameter as the suitable weighted parameter of the adjacency function. After calculation, we set soft-threshold β as 23 and selected a correlation coefficient close to 0.85 to construct gene modules (Figure 1A). In accordance with the basic idea of WGCNA, we determined the correlation matrix and adjacency matrix of the gene expression profile of all samples with the clinical features, and then converted them to a topological overlap matrix (TOM), which produced a system clustering tree of genes according to gene-gene non-ω similarity. Along with TOM, we performed hierarchical average linkage clustering to distinguish the gene modules of each gene network (deep split = 2, cut height = 0.25) (Figure 1C). In all samples, approximately eight gene modules were recognized in total by the dynamic tree cut (Figure 1B). The genes whose expression levels could not be correlated with clinical features were assigned to the gray module. Therefore, the genes within the gray module were not further analyzed. A heatmap of the topological overlap in the gene network is shown in Figure 1D.

### Finding Module of Interest

**Figure 2:**
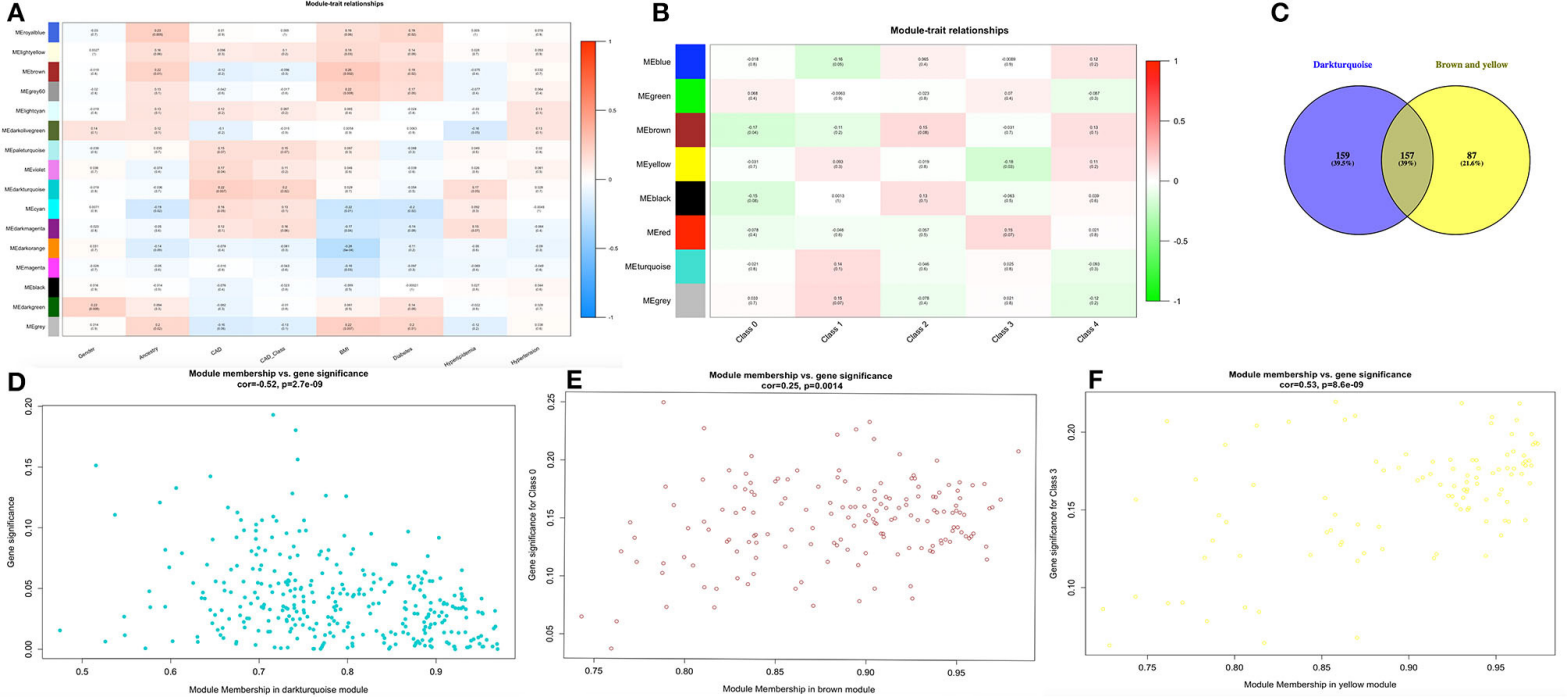
Finding module of interest. **(A,B)** Module-feature associations. Each row corresponds to a module eigengene, and each column corresponds to a clinical feature. Each cell contains the corresponding correlation in the first line and the *P*-value in the second line. The table is color-coded by correlation according to the color legend. **(C)** Venn diagram for these three modules. **(D–F)** Scatter plot of eigengene modules.

The heatmap showed numerous valued biological significance to identify modules with the most significant and associated clinical characteristics. As shown in Figure 2A, the most interesting associations in the module-feature relationship were the dark turquoise module and CAD (*r*^2^ = 0.22, *P* = 0.007) and the dark turquoise module and CAD_class (*r*^2^ = 0.2, *P* = 0.02). The dark turquoise module membership and gene significance were significantly negatively correlated (*r*^2^ = −0.52, *P* = 2.7E-09) (Figure 2D). Subsequently, we analyzed the relationship between the module and CAD class and found that the brown module and class 0 (*r*^2^ = −0.17, *P* = 0.04) and the yellow module and class 3 (*r*^2^ = −0.18, *P* = 0.03) had the most interesting associations (Figure 2B). The brown and yellow module membership and gene significance were significantly positively correlated (*r*^2^ = 0.25, *P* = 0.0014; *r*^2^ = 0.53, *P* = 8.6E-09, respectively) (Figures 2E,F). From the dark turquoise module, we identified 316 genes in total, and the brown and yellow modules had 244 genes in total. The details of the module genes are shown in Supplementary Tables 2, 3, and a Venn diagram is shown in Figure 2C. The other module did not have enough relationships or statistical significance for further consideration.

### Functional Annotation

**Figure 3:**
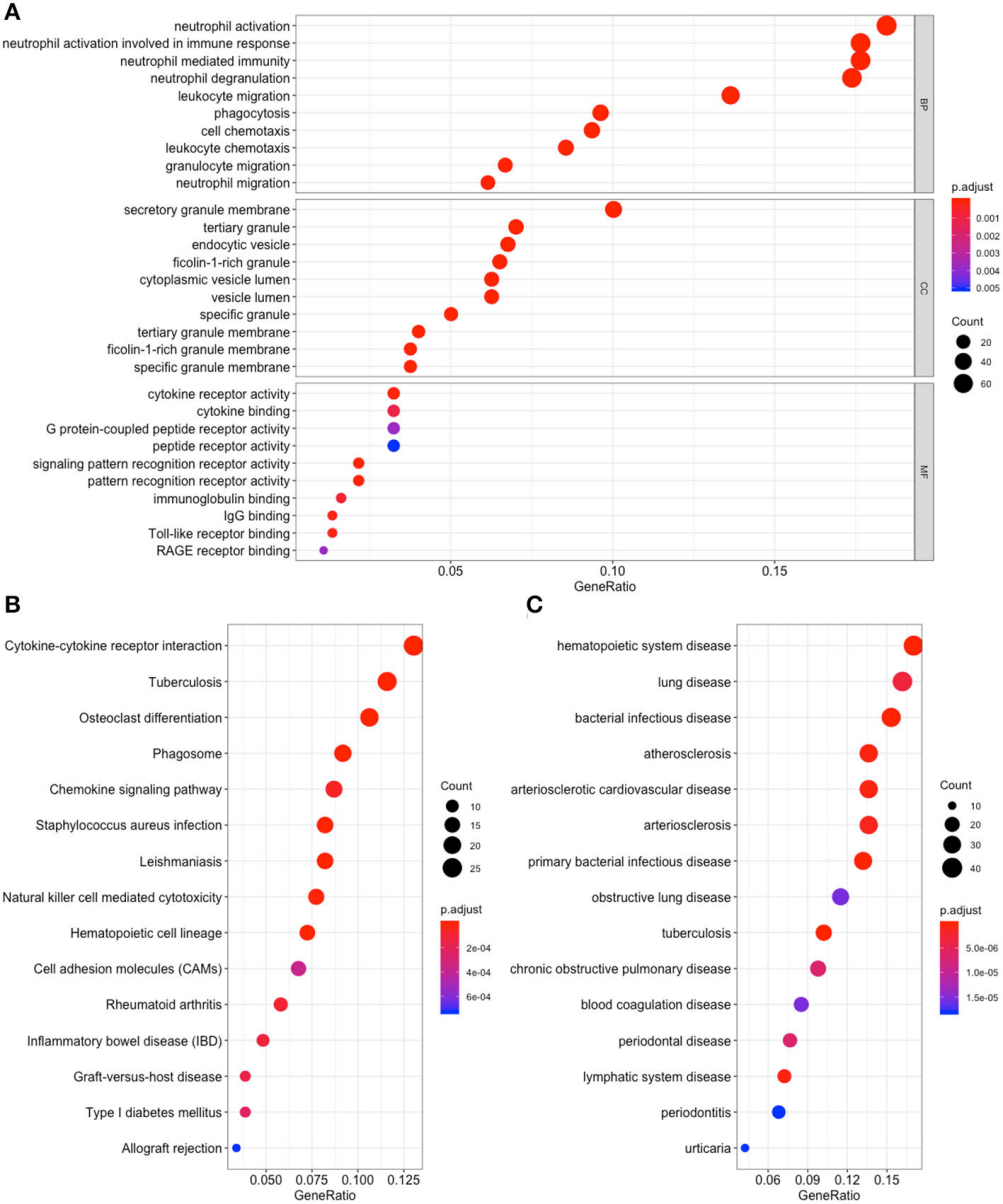
Functional enrichment for genes in the object module. The x-axis shows the ratio number of genes, and the y-axis shows the pathway terms. The –log10 (*P*-value) of each term is colored according to the legend. **(A)** Gene Ontology analysis; **(B)** Kyoto Encyclopedia of Genes and Genomes (KEGG) pathway analysis; **(C)** Disease Ontology analysis.

To detect the biological relevance of module genes and function, all three module genes were subjected to GO functional, KEGG pathway and DO enrichment analyses. The results are shown in Figure 3. When analyzing GO functions, we identified 484 biological processes, 39 cellular components, and 22 molecular functions with an adjusted *P* < 0.05. Table 1 shows the top 10 items. In total, 38 pathways were enriched in KEGG pathway analysis, and 147 DO items with an adjusted *P* < 0.05 (false discovery rate, FDR set at < 0.05) were identified. Table 2 shows the top 15 items.

**Table 1:** GO analysis for genes (top 10 significantly enriched terms).

**Item**	**ID**	**Description**	***P*. adjust**	**geneID**
BP	GO:0042119	Neutrophil activation	2.94E-34	ALOX5/ARHGAP9/BIN2/BST1/CCL5/CD14/CD53/CFP/CXCR1/CXCR2/FCER1G/FCGR3B/FCN1/FGL2/FGR/FPR1/FPR2/HBB/HSPA6/HVCN1/IL18RAP/ITGAM/ITGAX/ITGB2/LILRA2/LILRA3/LILRB2/LILRB3/MCEMP1/MGAM/MMP25/MNDA/OSCAR/PECAM1/PPBP/PRAM1/PTAFR/PTPN6/RAB37/S100A12/S100A8/S100A9/SIGLEC5/SIRPB1/SLC11A1/STK10/TLR2/TNFRSF1B/TYROBP/ADAM8/ADGRE3/C5AR1/CD300A/CLEC4D/CTSS/FCGR2A/HSP90AB1/ITGAL/KMT2E/LRMP/MMP9/PGLYRP1/PLEKHO2/PRKCD/RAB24/SELL/SPTAN1/SYK/TBC1D10C
BP	GO:0002283	Neutrophil activation involved in immune response	3.13E-32	ALOX5/ARHGAP9/BIN2/BST1/CD14/CD53/CFP/CXCR1/CXCR2/FCER1G/FCGR3B/FCN1/FGL2/FGR/FPR1/FPR2/HBB/HSPA6/HVCN1/ITGAM/ITGAX/ITGB2/LILRA2/LILRA3/LILRB2/LILRB3/MCEMP1/MGAM/MMP25/MNDA/OSCAR/PECAM1/PPBP/PRAM1/PTAFR/PTPN6/RAB37/S100A12/S100A8/S100A9/SIGLEC5/SIRPB1/SLC11A1/STK10/TLR2/TNFRSF1B/TYROBP/ADAM8/ADGRE3/C5AR1/CD300A/CLEC4D/CTSS/FCGR2A/HSP90AB1/ITGAL/LRMP/MMP9/PGLYRP1/PLEKHO2/PRKCD/RAB24/SELL/SPTAN1/SYK/TBC1D10C
BP	GO:0002446	Neutrophil mediated immunity	8.39E-32	ALOX5/ARHGAP9/BIN2/BST1/CD14/CD53/CFP/CXCR1/CXCR2/FCER1G/FCGR3B/FCN1/FGL2/FGR/FPR1/FPR2/HBB/HSPA6/HVCN1/ITGAM/ITGAX/ITGB2/LILRA3/LILRB2/LILRB3/MCEMP1/MGAM/MMP25/MNDA/OSCAR/PECAM1/PPBP/PRAM1/PTAFR/PTPN6/RAB37/S100A12/S100A8/S100A9/SIGLEC5/SIRPB1/SLC11A1/STK10/TLR2/TNFRSF1B/TYROBP/ADAM8/ADGRE3/C5AR1/CD300A/CLEC4D/CTSS/FCGR2A/HSP90AB1/ITGAL/KMT2E/LRMP/MMP9/PGLYRP1/PLEKHO2/PRKCD/RAB24/SELL/SPTAN1/SYK/TBC1D10C
BP	GO:0043312	Neutrophil degranulation	9.61E-32	ALOX5/ARHGAP9/BIN2/BST1/CD14/CD53/CFP/CXCR1/CXCR2/FCER1G/FCGR3B/FCN1/FGL2/FGR/FPR1/FPR2/HBB/HSPA6/HVCN1/ITGAM/ITGAX/ITGB2/LILRA3/LILRB2/LILRB3/MCEMP1/MGAM/MMP25/MNDA/OSCAR/PECAM1/PPBP/PRAM1/PTAFR/PTPN6/RAB37/S100A12/S100A8/S100A9/SIGLEC5/SIRPB1/SLC11A1/STK10/TLR2/TNFRSF1B/TYROBP/ADAM8/ADGRE3/C5AR1/CD300A/CLEC4D/CTSS/FCGR2A/HSP90AB1/ITGAL/LRMP/MMP9/PGLYRP1/PLEKHO2/PRKCD/RAB24/SELL/SPTAN1/SYK/TBC1D10C
BP	GO:0050900	Leukocyte migration	9.49E-20	BST1/CCL4/CCL5/CCR7/CORO1A/CSF3R/CXCR1/CXCR2/DOK2/DYSF/FCER1G/FFAR2/FPR2/HCK/IL16/ITGAM/ITGAX/ITGB2/JAML/PECAM1/PF4/PPBP/PTAFR/PTPN6/S100A12/S100A8/S100A9/SELPLG/SIRPG/SLC7A7/STK10/TBX21/TNFSF14/TREM1/ADAM8/C5AR1/CD2/CD244/CD300A/CXCR3/DAPK2/GPSM3/IL17RA/IL1B/IL6R/ITGAL/LYN/MMP9/SELL/SYK/VAV1
BP	GO:0030595	Leukocyte chemotaxis	7.16E-16	BST1/CCL4/CCL5/CCR7/CORO1A/CSF3R/CXCR1/CXCR2/DYSF/FCER1G/FFAR2/FPR2/IL16/ITGB2/JAML/PF4/PPBP/S100A12/S100A8/S100A9/TNFSF14/ADAM8/C5AR1/CXCR3/DAPK2/GPSM3/IL17RA/IL1B/IL6R/LYN/SYK/VAV1
BP	GO:0097530	Granulocyte migration	1.31E-14	BST1/CCL4/CCL5/CCR7/CSF3R/CXCR1/CXCR2/DYSF/FCER1G/ITGB2/JAML/PECAM1/PF4/PPBP/S100A12/S100A8/S100A9/ADAM8/C5AR1/CD300A/DAPK2/IL17RA/IL1B/SYK/VAV1
BP	GO:0060326	Cell chemotaxis	1.45E-14	BIN2/BST1/CCL4/CCL5/CCR7/CORO1A/CSF3R/CXCR1/CXCR2/DYSF/FCER1G/FFAR2/FPR2/IL16/ITGB2/JAML/PF4/PPBP/S100A12/S100A8/S100A9/TNFSF14/ADAM8/ARRB2/C5AR1/CXCR3/DAPK2/GPSM3/IL17RA/IL1B/IL6R/LYN/PRKCD/SYK/VAV1
BP	GO:1990266	Neutrophil migration	2.12E-14	BST1/CCL4/CCL5/CCR7/CSF3R/CXCR1/CXCR2/DYSF/FCER1G/ITGB2/JAML/PECAM1/PF4/PPBP/S100A12/S100A8/S100A9/ADAM8/C5AR1/DAPK2/IL1B/SYK/VAV1
BP	GO:0006909	Phagocytosis	1.90E-13	ARHGAP25/BIN2/CD14/CD300LF/CORO1A/DYSF/FCER1G/FCGR3A/FCN1/FGR/HCK/ITGAM/ITGB2/MARCO/NCF2/NCF4/PECAM1/SIRPB1/SIRPG/SLC11A1/TLR2/TMEM175/WAS/CD300A/CEACAM4/FCGR2A/HSP90AB1/IL1B/ITGAL/LYN/NCK1/PLCG2/PRKCD/SYK/VAV1/WIPF1
CC	GO:0030667	Secretory granule membrane	5.17E-19	BST1/CD14/CD53/CXCR1/CXCR2/FCER1G/FCGR3B/FPR1/FPR2/HVCN1/ITGAM/ITGAX/ITGB2/LILRA3/LILRB2/LILRB3/MCEMP1/MGAM/MMP25/PECAM1/PTAFR/RAB37/SIGLEC5/SIRPB1/SLC11A1/STK10/TLR2/TNFRSF1B/TYROBP/ADAM8/ADGRE3/C5AR1/CD300A/CLEC4D/FCGR2A/ITGAL/LRMP/RAB24/SELL/TBC1D10C
CC	GO:0070820	Tertiary granule	7.56E-16	CD53/CFP/FCER1G/FPR1/FPR2/HBB/ITGAM/ITGAX/ITGB2/LILRA3/LILRB2/MCEMP1/MGAM/OSCAR/PPBP/PTAFR/PTPN6/SIGLEC5/SLC11A1/ADAM8/ADGRE3/CD300A/CLEC4D/CTSS/MMP9/PGLYRP1/SPTAN1/TBC1D10C
CC	GO:0101002	Ficolin-1-rich granule	1.07E-12	ALOX5/BIN2/FCER1G/FCN1/FGL2/FPR1/FPR2/HBB/HSPA6/ITGAX/ITGB2/LILRA3/LILRB2/MGAM/MNDA/SIGLEC5/SLC11A1/ADAM8/ADGRE3/CD300A/CLEC4D/CTSS/HSP90AB1/MMP9/PLEKHO2/TBC1D10C
CC	GO:0101003	Ficolin-1-rich granule membrane	9.10E-11	FCER1G/FPR1/FPR2/ITGAX/ITGB2/LILRA3/LILRB2/MGAM/SIGLEC5/SLC11A1/ADAM8/ADGRE3/CD300A/CLEC4D/TBC1D10C
CC	GO:0070821	Tertiary granule membrane	9.10E-11	CD53/FCER1G/FPR2/ITGAM/ITGAX/ITGB2/LILRA3/LILRB2/MCEMP1/MGAM/PTAFR/SIGLEC5/SLC11A1/ADAM8/CD300A/CLEC4D
CC	GO:0030139	Endocytic vesicle	4.36E-09	CORO1A/DYSF/FCGR1B/GNLY/HBB/HLA-DRA/HLA-DRB4/HLA-DRB5/MARCO/NCF1/NCF2/NCF4/SLC11A1/TLR2/WAS/ADAM8/ARRB2/CD163/DVL2/HLA-DRB1/HLA-DRB3/LPAR2/PGLYRP1/RAB11FIP1/RAB24/SYK/TLR1
CC	GO:0042581	Specific granule	5.40E-09	BST1/CD53/CFP/FPR2/HVCN1/ITGAM/ITGB2/LILRA3/MCEMP1/MMP25/OSCAR/PTPN6/RAB37/STK10/TNFRSF1B/ADAM8/CLEC4D/ITGAL/PGLYRP1/SPTAN1
CC	GO:0035579	Specific granule membrane	2.13E-08	BST1/CD53/FPR2/HVCN1/ITGAM/ITGB2/LILRA3/MCEMP1/MMP25/RAB37/STK10/TNFRSF1B/ADAM8/CLEC4D/ITGAL
CC	GO:0060205	Cytoplasmic vesicle lumen	1.28E-06	ALOX5/ARHGAP9/BIN2/CFP/DEFA3/F13A1/FCN1/FGR/GNLY/HBB/HSPA6/MNDA/OSCAR/PF4/PPBP/PTPN6/S100A12/S100A8/S100A9/SRGN/GHRL/HSP90AB1/PGLYRP1/PRKCD/SPTAN1
CC	GO:0031983	Vesicle lumen	1.28E-06	ALOX5/ARHGAP9/BIN2/CFP/DEFA3/F13A1/FCN1/FGR/GNLY/HBB/HSPA6/MNDA/OSCAR/PF4/PPBP/PTPN6/S100A12/S100A8/S100A9/SRGN/GHRL/HSP90AB1/PGLYRP1/PRKCD/SPTAN1
MF	GO:0008329	Signaling pattern recognition receptor activity	1.07E-06	CD14/FCN1/MARCO/PTAFR/TLR2/TLR8/PGLYRP1/TLR4
MF	GO:0038187	Pattern recognition receptor activity	1.07E-06	CD14/FCN1/MARCO/PTAFR/TLR2/TLR8/PGLYRP1/TLR4
MF	GO:0004896	Cytokine receptor activity	9.91E-05	CCR7/CSF3R/CXCR1/CXCR2/IL18RAP/IL1R2/IL2RB/CSF2RA/CXCR3/IL10RA/IL17RA/IL6R
MF	GO:0019864	IgG binding	0.00020022	FCER1G/FCGR1B/FCGR3A/FCGR3B/FCGR2A
MF	GO:0035325	Toll-like receptor binding	0.00020022	S100A8/S100A9/TLR2/SYK/TLR1
MF	GO:0019865	Immunoglobulin binding	0.00061902	FCER1G/FCGR1B/FCGR3A/FCGR3B/LILRA2/FCGR2A
MF	GO:0019955	Cytokine binding	0.00110874	CCR7/CSF1R/CSF3R/CXCR1/CXCR2/IL1R2/IL2RB/TNFRSF1B/CSF2RA/CXCR3/IL10RA/IL6R
MF	GO:0050786	RAGE receptor binding	0.00380145	FPR1/S100A12/S100A8/S100A9
MF	GO:0008528	G protein-coupled peptide receptor activity	0.00380145	CCR7/CXCR1/CXCR2/FPR1/FPR2/GALR3/KISS1R/OGFR/UTS2R/CXCR3/INPP5K/SIGMAR1
MF	GO:0001653	Peptide receptor activity	0.00507011	CCR7/CXCR1/CXCR2/FPR1/FPR2/GALR3/KISS1R/OGFR/UTS2R/CXCR3/INPP5K/SIGMAR1

*GO, gene ontology; BP, biological processes; CC, cellular components; MF, molecular functions*.

**Table 2:** KEGG and DO analysis for genes (top 15 significantly enriched terms).

**Item**	**ID**	**Description**	***P*. adjust**	**geneID**
KEGG	hsa05150	*Staphylococcus aureus* infection	1.72E-10	DEFA3/FCGR3A/FCGR3B/FPR1/FPR2/HLA-DRA/HLA-DRB4/HLA-DRB5/ITGAM/ITGB2/PTAFR/SELPLG/C5AR1/FCGR2A/HLA-DRB1/HLA-DRB3/ITGAL
KEGG	hsa04380	Osteoclast differentiation	1.72E-10	CSF1R/FCGR3A/FCGR3B/LCP2/LILRA2/LILRA3/LILRA4/LILRB2/LILRB3/NCF1/NCF2/NCF4/OSCAR/SIRPB1/SIRPG/TYROBP/FCGR2A/IL1B/LILRA5/PLCG2/SPI1/SYK
KEGG	hsa05140	Leishmaniasis	3.34E-10	FCGR3A/FCGR3B/HLA-DRA/HLA-DRB4/HLA-DRB5/ITGAM/ITGB2/NCF1/NCF2/NCF4/PTPN6/TLR2/FCGR2A/HLA-DRB1/HLA-DRB3/IL1B/TLR4
KEGG	hsa05152	Tuberculosis	1.98E-09	BCL2/CD14/CLEC4E/CORO1A/FCER1G/FCGR3A/FCGR3B/HLA-DRA/HLA-DRB4/HLA-DRB5/ITGAM/ITGAX/ITGB2/TLR2/CTSS/FCGR2A/HLA-DRB1/HLA-DRB3/IL10RA/IL1B/LSP1/SYK/TLR1/TLR4
KEGG	hsa04060	Cytokine-cytokine receptor interaction	5.06E-07	BMP8B/CCL4/CCL5/CCR7/CSF1R/CSF3R/CXCR1/CXCR2/IL16/IL18RAP/IL1R2/IL2RB/LTB/OSM/PF4/PPBP/TNFRSF10C/TNFRSF1B/TNFSF14/CD27/CSF2RA/CXCR3/IL10RA/IL17RA/IL1B/IL6R/TNFSF10
KEGG	hsa04145	Phagosome	5.32E-07	CD14/CORO1A/FCGR3A/FCGR3B/HLA-DRA/HLA-DRB4/HLA-DRB5/ITGAM/ITGB2/MARCO/NCF1/NCF2/NCF4/TLR2/CTSS/FCGR2A/HLA-DRB1/HLA-DRB3/TLR4
KEGG	hsa04640	Hematopoietic cell lineage	8.96E-07	CD14/CD37/CSF1R/CSF3R/HLA-DRA/HLA-DRB4/HLA-DRB5/IL1R2/ITGAM/CD2/CSF2RA/HLA-DRB1/HLA-DRB3/IL1B/IL6R
KEGG	hsa04650	Natural killer cell mediated cytotoxicity	7.99E-06	FCER1G/FCGR3A/FCGR3B/GZMB/HCST/ITGB2/LCP2/PRF1/PTPN6/TYROBP/CD244/ITGAL/PLCG2/SYK/TNFSF10/VAV1
KEGG	hsa04062	Chemokine signaling pathway	5.75E-05	CCL4/CCL5/CCR7/CXCR1/CXCR2/FGR/HCK/NCF1/PF4/PPBP/RASGRP2/WAS/ARRB2/CXCR3/LYN/PLCB2/PRKCD/VAV1
KEGG	hsa05323	Rheumatoid arthritis	9.05E-05	CCL5/HLA-DRA/HLA-DRB4/HLA-DRB5/ITGB2/LTB/TLR2/HLA-DRB1/HLA-DRB3/IL1B/ITGAL/TLR4
KEGG	hsa05321	Inflammatory bowel disease (IBD)	0.00013055	HLA-DRA/HLA-DRB4/HLA-DRB5/IL18RAP/TBX21/TLR2/HLA-DRB1/HLA-DRB3/IL1B/TLR4
KEGG	hsa05332	Graft-vs.-host disease	0.00016688	GZMB/HLA-DRA/HLA-DRB4/HLA-DRB5/PRF1/HLA-DRB1/HLA-DRB3/IL1B
KEGG	hsa04940	Type I diabetes mellitus	0.0002234	GZMB/HLA-DRA/HLA-DRB4/HLA-DRB5/PRF1/HLA-DRB1/HLA-DRB3/IL1B
KEGG	hsa04514	Cell adhesion molecules (CAMs)	0.00037834	CADM3/HLA-DRA/HLA-DRB4/HLA-DRB5/ITGAM/ITGB2/PECAM1/SELPLG/CD2/HLA-DRB1/HLA-DRB3/ITGAL/NTNG2/SELL
KEGG	hsa05330	Allograft rejection	0.00072889	GZMB/HLA-DRA/HLA-DRB4/HLA-DRB5/PRF1/HLA-DRB1/HLA-DRB3
DO	DOID:104	Bacterial infectious disease	7.86E-12	ALOX5/BCL2/CCL4/CCL5/CCR7/CD14/CXCR2/DEFA3/FPR2/GZMA/HLA-DRB4/ITGAM/ITGAX/ITGB2/NLRP3/OSM/PECAM1/PRF1/PTAFR/SLC11A1/TLR2/TNFRSF1B/TREM1/C5AR1/CD27/FCGR2A/GHRL/HLA-DRB1/IL1B/ITGAL/MCL1/MMP9/SELL/TLR1/TLR4/TP53
DO	DOID:0050338	Primary bacterial infectious disease	5.63E-10	ALOX5/BCL2/CCL4/CCL5/CCR7/CD14/CXCR2/DEFA3/GZMA/HLA-DRB4/ITGAX/NLRP3/PECAM1/PRF1/SLC11A1/TLR2/TNFRSF1B/TREM1/C5AR1/CD27/FCGR2A/GHRL/HLA-DRB1/IL1B/ITGAL/MCL1/MMP9/SELL/TLR1/TLR4/TP53
DO	DOID:399	Tuberculosis	4.17E-09	ALOX5/BCL2/CCL4/CCL5/CD14/CXCR2/DEFA3/GZMA/ITGAX/NLRP3/PECAM1/PRF1/SLC11A1/TLR2/TNFRSF1B/CD27/HLA-DRB1/IL1B/ITGAL/MCL1/MMP9/TLR1/TLR4/TP53
DO	DOID:74	Hematopoietic system disease	1.49E-07	BCAM/BCL2/CCL5/CSF3R/CXCR1/F13A1/FCGR3A/FCGR3B/HBB/HLA-DRA/ITGAM/ITGB2/PF4/PRF1/PTPN6/RASGRP4/SLC11A1/TBX21/TLR2/TLR8/TNFRSF1B/WAS/ZFPM1/CSF2RA/CXCR3/DCK/ETV6/FCGR2A/HLA-DRB1/HLA-DRB3/IL1B/MMP9/SELL/SPTAN1/TINF2/TLR4/TNFSF10/TP53/VAV1/WIPF1
DO	DOID:75	Lymphatic system disease	5.22E-07	CCR7/FOXC2/GZMB/ITGAX/PECAM1/PRF1/S100A12/S100A8/S100A9/SLC11A1/CD163/HLA-DRB1/IL1B/ITGAL/MMP9/TLR4/TP53
DO	DOID:1936	Atherosclerosis	5.22E-07	ALOX5/ALOX5AP/APOBR/CCL5/CD14/CXCR2/FCGR3A/FPR1/MNDA/NLRP3/PECAM1/PF4/S100A12/S100A8/S100A9/SOX18/TLR2/UTS2R/ADAM8/ADRB2/CD163/CTSS/CXCR3/FCGR2A/GHRL/IL1B/KLF2/MMP9/PGLYRP1/TLR4/TNFSF10/TP53
DO	DOID:2348	Arteriosclerotic cardiovascular disease	5.22E-07	ALOX5/ALOX5AP/APOBR/CCL5/CD14/CXCR2/FCGR3A/FPR1/MNDA/NLRP3/PECAM1/PF4/S100A12/S100A8/S100A9/SOX18/TLR2/UTS2R/ADAM8/ADRB2/CD163/CTSS/CXCR3/FCGR2A/GHRL/IL1B/KLF2/MMP9/PGLYRP1/TLR4/TNFSF10/TP53
DO	DOID:2349	Arteriosclerosis	9.86E-07	ALOX5/ALOX5AP/APOBR/CCL5/CD14/CXCR2/FCGR3A/FPR1/MNDA/NLRP3/PECAM1/PF4/S100A12/S100A8/S100A9/SOX18/TLR2/UTS2R/ADAM8/ADRB2/CD163/CTSS/CXCR3/FCGR2A/GHRL/IL1B/KLF2/MMP9/PGLYRP1/TLR4/TNFSF10/TP53
DO	DOID:850	Lung disease	3.13E-06	BCL2/CCL5/CD14/CSTA/CXCR1/FCGR3B/FGL2/GZMA/HBB/HCK/IL16/IL18RAP/ITGAM/ITGB2/NCF1/NCF2/S100A9/SELPLG/SLC11A1/TBX21/TLR2/TREM1/ADAM8/ADRB2/C5AR1/CSF2RA/CTSS/CXCR3/FCGR2A/HLA-DRB1/IL10RA/IL1B/ITGAL/MMP9/NLRP1/PRKCD/TLR4/TP53
DO	DOID:3083	Chronic obstructive pulmonary disease	6.69E-06	BCL2/CCL5/CD14/CSTA/FGL2/GZMA/HBB/HCK/IL16/ITGAM/ITGB2/SLC11A1/TLR2/TREM1/ADRB2/C5AR1/CTSS/CXCR3/IL10RA/IL1B/ITGAL/MMP9/TLR4
DO	DOID:3388	Periodontal disease	6.69E-06	CCL5/CD14/CXCR2/DEFA3/FCGR3A/FCGR3B/FPR1/IL16/IL1R2/OSM/PECAM1/S100A8/TLR2/FCGR2A/IL1B/MMP9/TLR4/TNFSF10
DO	DOID:1247	Blood coagulation disease	1.55E-05	CCL5/F13A1/FCGR3B/HLA-DRA/ITGAM/ITGB2/PF4/PRF1/TLR2/WAS/ZFPM1/FCGR2A/HLA-DRB1/HLA-DRB3/IL1B/MMP9/SELL/TLR4/TNFSF10/WIPF1
DO	DOID:2320	Obstructive lung disease	1.56E-05	BCL2/CCL5/CD14/CSTA/FGL2/GZMA/HBB/HCK/IL16/IL18RAP/ITGAM/ITGB2/SELPLG/SLC11A1/TBX21/TLR2/TREM1/ADAM8/ADRB2/C5AR1/CTSS/CXCR3/IL10RA/IL1B/ITGAL/MMP9/TLR4
DO	DOID:824	Periodontitis	1.81E-05	CCL5/CD14/CXCR2/DEFA3/FCGR3A/FCGR3B/FPR1/IL16/IL1R2/OSM/S100A8/TLR2/FCGR2A/IL1B/MMP9/TLR4
DO	DOID:1555	Urticaria	1.81E-05	ALOX5/ALOX5AP/FPR2/NLRP3/PF4/PPBP/ADRB2/HLA-DRB1/MMP9/SYK

*DO, disease ontology; KEGG, Kyoto Encyclopedia of Genes and Genome pathway enrichment analyses*.

Among these results, several items have been confirmed in previous studies that were associated with CAD, and these included Neutrophil degranulation (GO:0043312); regulation of MAP kinase activity (GO:0043405); Neutrophil activation involved in immune response (GO:0002283); Neutrophil mediated immunity (GO:0002446); Neutrophil activation (GO:0042119); Atherosclerosis (DOID:2349); Atherosclerosis (DOID:1936); Atherosclerotic cardiovascular disease (DOID:2348); Cell adhesion molecules (CAMs) (hsa04514); Cytokine -cytokine receptor interaction (hsa04060); Fluid shear stress and atherosclerosis (hsa05418); Rheumatoid arthritis (hsa05323); and NF-kappa B signaling pathway (hsa04064). The genes located in these items were selected for further analysis.

### PPI Network Construction and Identification of Hub Genes

**Figure 4:**
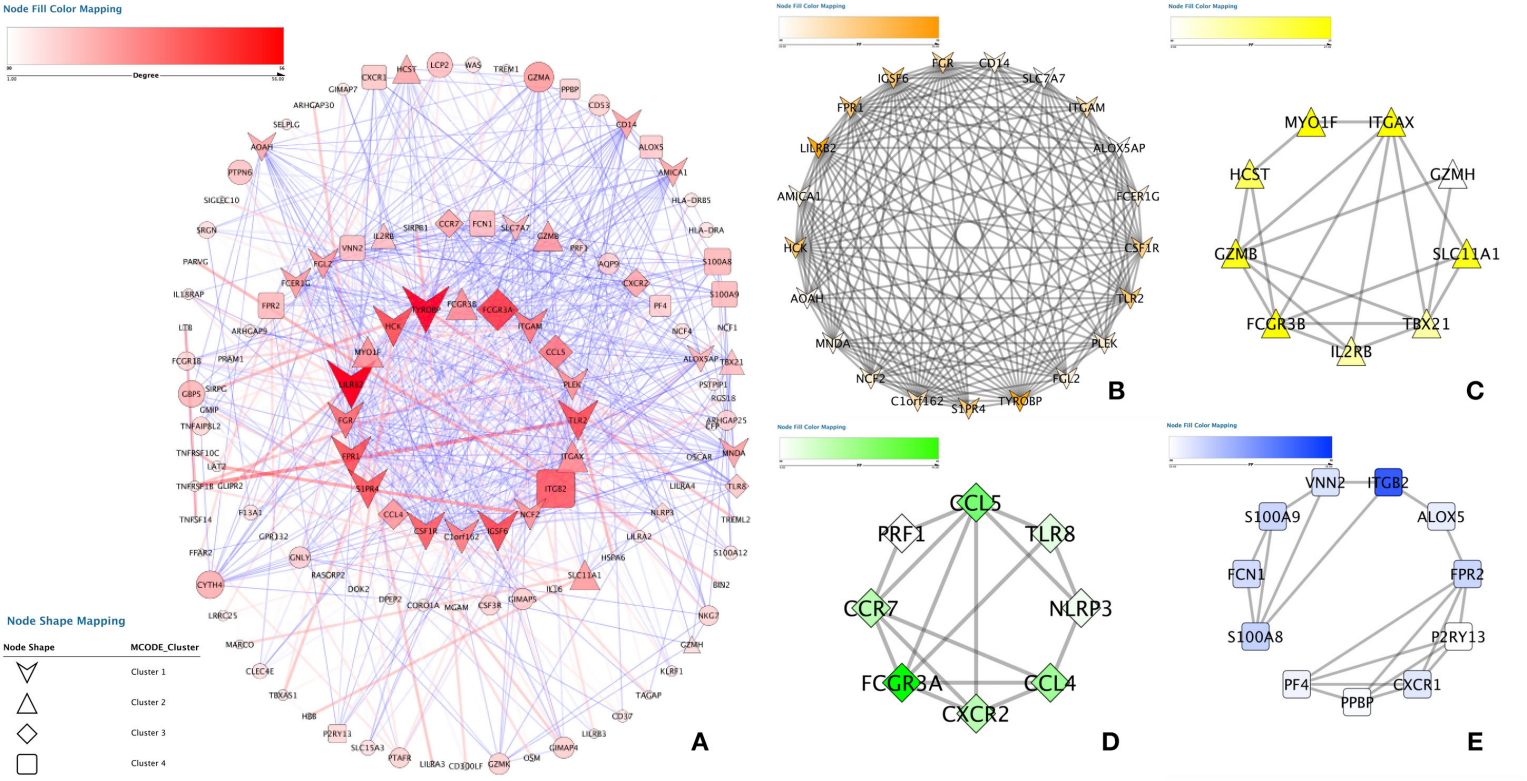
Protein-protein interaction analysis. **(A)** Protein–protein interaction network of the module genes. Edges represent the interaction between two genes. A degree was used to describe the importance of protein nodes in the network; red shows a high degree, and green presents a low degree. **(B–E)** The significant modules identified from the protein-protein interaction network using the molecular complex detection method with a score of > 8.0. MCODE_B_ score = 12.537, MCODE_c_ score = 11.4486, MCODE_D_ score = 9.301 and MCODE_E_ score = 8.671.

To elucidate the PPI network of these selected genes, we used the STRING database to perform the analysis. When the cutoff was set as a combined score > 0.9, in total, 3,218 protein pairs and 285 nodes were included. Figure 4A shows the net analysis from Cytoscape. Four modules with a score > 10 were found and are presented in Figures 4B–E for detection using the Molecular Complex Detection (MCODE) app.

These four modules included 49 genes in total. Finally, approximately six genes demonstrated a high degree of association simultaneously in the submodule analysis and were screened with GO, DO, and KEGG data. These six genes were toll-like receptor 2 (TLR2), CD14 molecule (CD14), NLR family pyrin domain containing 3 (NLRP3), C-X-C motif chemokine receptor 2 (CXCR2), C-C motif chemokine ligand 5 (CCL5) and platelet factor 4 (PF4).

**Figure 5:**
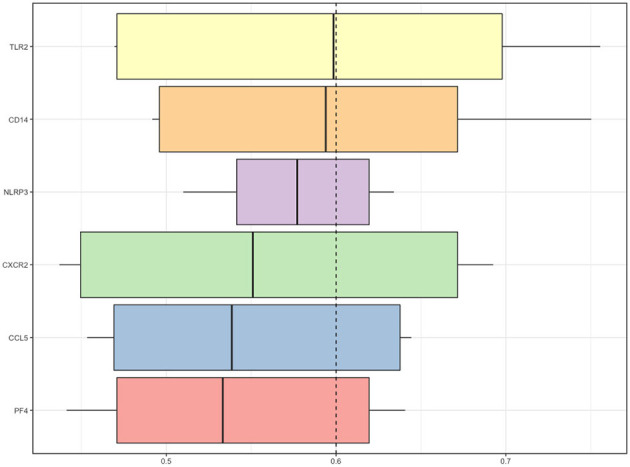
Summary of functional similarities. The distributions of functional similarities are summarized as boxplots. The boxes represent the middle 50% of the similarities. The lines in the boxes indicate the mean of the functional similarities. Proteins with a higher average functional similarity were defined as party proteins, which are considered central proteins. The dashed line represents the cutoff value.

We employed the GoSemSim package in R to analyze the interactions between the proteins and scored them according to their average functional similarity. Proteins with higher scores had higher functional interactions and warranted further investigation. *TLR2* and *CD14* were the two top-ranked proteins potentially playing central roles in the interaction (Figure 5).

**Figure 6:**
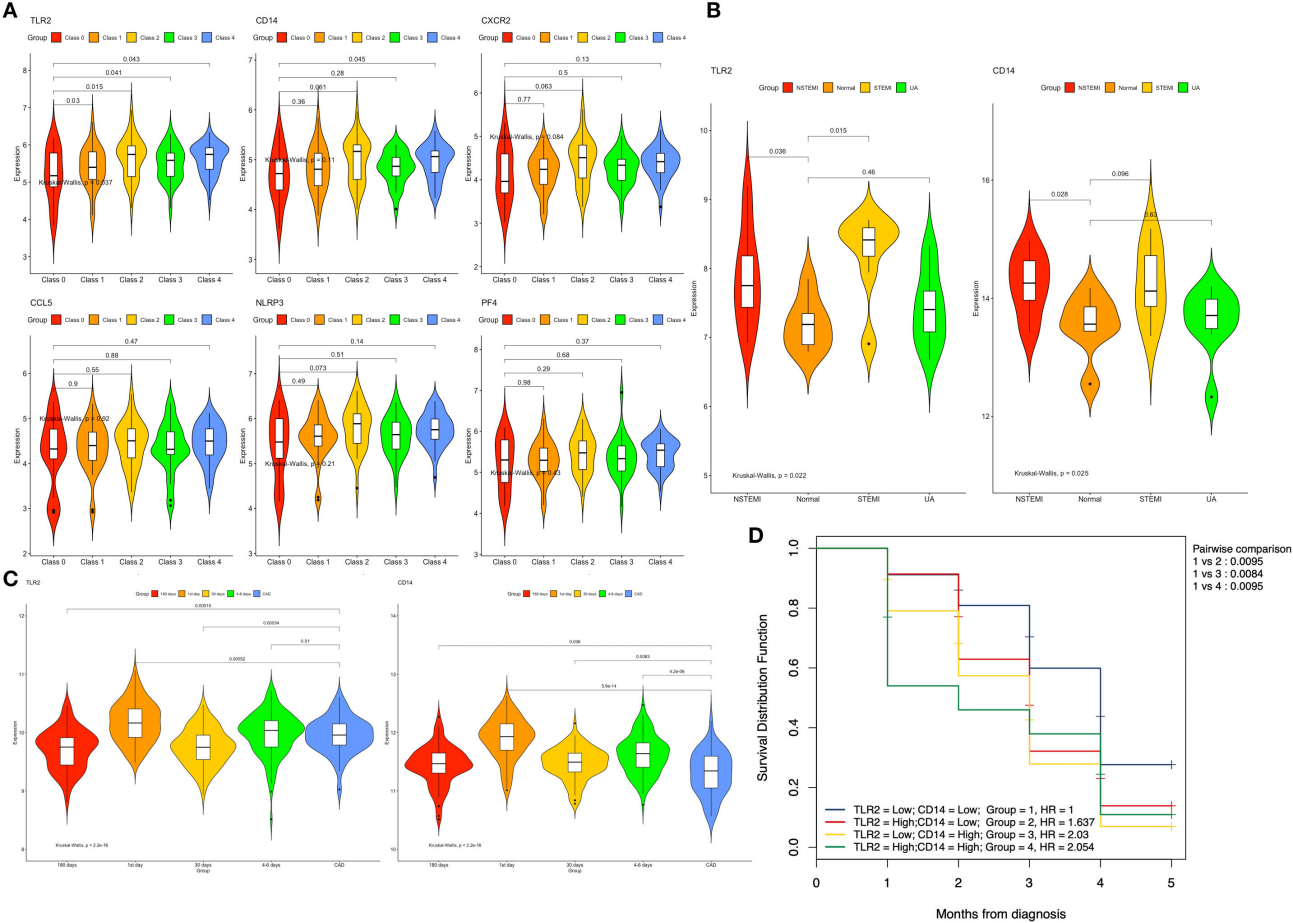
Expression of the hub genes in different datasets. **(A)** Expression of the six genes among different classes of coronary arteries in GSE90074. **(B)** Expression of the *TLR2* and *CD14* genes between patients with different types of acute coronary syndrome and normal controls in GSE60993. **(C)** Expression of the *TLR2* and *CD14* genes between different time points after myocardial infarction and stable coronary artery disease in GSE59867. **(D)** Overall survival curves using combinations of *TLR2* and *CD14* expression levels in GSE59867. Univariate Cox regression was used to determine the HR, and the log rank *P*-values is reported; Bonferroni multiple testing adjustment was used for pairwise comparisons (*P* = 0.05/5 = 0.01). HR, Hazard ratio.

At the same time, we found that the expression of *TLR2* was higher in different classes of coronary artery lesions than in class 0, while the expression of *CD14* was higher only in class 4 than in class 0. The expression of the other genes was not significantly different in all groups (Figure 6A). This result suggests that *TLR2* and *CD14* may be related to the severity of coronary atherosclerosis.

### Hub Gene Validation

The training data set (GSE60993) was used to verify the correlation between two hub genes (*TLR2* and *CD14*) and atherosclerotic plaque vulnerability. We compared the expression of each candidate hub gene during the development of CAD (including stable CAD, unstable angina, NSTEMI and STEMI) (Table 3). One-way analysis of variance test results showed that the expression of *TLR2* was closely related to NSTEMI and STEMI, whereas the expression of *CD14* was only closely related to NSTEMI. Patients with acute myocardial infarction had a high level of gene expression (Figure 6B).

**Table 3:** Comparison of demographic, lifestyle characteristics and serum lipid levels among different groups.

**Parameter**	**Control**	**CAD**	**UA**	**NSTEMI**	**STEMI**
Number	212	220	204	191	202
Male/female	44/174	39/181	44/160	34/157	48/154
Age (years)^1^	55.27 ± 8.24	54.29 ± 8.43	55.57 ± 7.92	56.11 ± 8.91	56.02 ± 7.78
Height (cm)	166.13 ± 6.74	167.63 ± 7.10	167.49 ± 7.26	166.52 ± 6.88	167.09 ± 8.74
Weight (kg)	52.46 ± 6.88	54.72 ± 9.93	58.61 ± 8.21^a^	57.89 ± 9.13^a^	58.12 ± 9.32^a^
Body mass index (kg/m^2^)	29.21 ± 5.23	30.24 ± 6.57^a^	30.87 ± 7.32^a^	30.89 ± 7.14^a^	30.96 ± 6.19^a^
Waist circumference (cm)	74.29 ± 6.71	73.54 ± 9.28	74.63 ± 7.65	75.28 ± 7.22	76.25 ± 6.31
Smoking status [*n* (%)]	56 (26.0)	79 (35.7)^a^	93 (45.8)^a^	92 (48.4)^a^	99 (48.9)^a^
Alcohol consumption [*n* (%)]	51 (24.2)	57 (25.7)	48 (23.3)	56 (29.2)^a^	58 (28.8)^a^
SBP (mmHg)	123.15 ± 16.22	129.32 ± 22.16^a^	148.01 ± 23.77^c^	141.45 ± 23.14^b^	106.45 ± 10.16^c^
DBP (mmHg)	81.54 ± 11.23	81.34 ± 12.01	89.04 ± 15.21^a^	83.23 ± 8.20	62.51 ± 18.74^c^
PP (mmHg)	49.64 ± 14.13	51.42 ± 13.59	51.66 ± 15.24	50.84 ± 15.22	50.22 ± 14.21
Glucose (mmol/L)	5.93 ± 1.81	6.22 ± 2.23	7.74 ± 2.21^b^	8.55 ± 2.31^c^	8.44 ± 2.69^c^
TC (mmol/L)	4.95 ± 1.32	5.26 ± 1.28^a^	5.69 ± 1.02^a^	5.99 ± 1.24^a^	5.84 ± 1.46^a^
TG (mmol/L)^2^	1.48 (0.51)	1.52 (1.21)	1.53 (1.22)	1.46 (1.41)	1.46 (1.38)
HDL-C (mmol/L)	1.51 ± 0.43	1.29 ± 0.34^a^	1.31 ± 0.31^a^	1.40 ± 0.31^a^	1.43 ± 0.35
LDL-C (mmol/L)	2.87 ± 0.83	3.24 ± 0.75^a^	3.99 ± 0.81^a^	3.89 ± 0.98^a^	3.96 ± 0.81^a^
ApoA1 (g/L)	1.23 ± 0.24	1.16 ± 0.22	1.18 ± 0.26	1.14 ± 0.26	1.15 ± 0.28
ApoB (g/L)	0.84 ± 0.19	0.82 ± 0.32	0.81 ± 0.31	0.94 ± 0.31	0.88 ± 0.28
ApoA1/ApoB	1.68 ± 0.49	1.67 ± 0.56	1.65 ± 0.52	1.66 ± 0.63	1.68 ± 0.55
Heart rate (beats/minutes)	72.43 ± 10.22	72.36 ± 10.34	77.21 ± 10.54^a^	76.45 ± 9.32^a^	79.78 ± 10.25^a^
Creatinine, (μmol/L)	72.55 ± 12.21	71.38 ± 11.44	74.43 ± 11.42	77.55 ± 10.21	76.68 ± 12.84
Uric acid, (μmol/L)	280.96 ± 74.23	283.89 ± 72.32	278.88 ± 84.32	286.91 ± 82.32	284.76 ± 76.38
Troponin T, (μg/L)	0.01 ± 0.02	0.02 ± 0.02	0.02 ± 0.01	2.74 ± 3.93^c^	2.86 ± 6.28^c^
CK, (U/L)	87.82 ± 43.21	88.84 ± 47.31	92.81 ± 51.32	1,211.93 ± 688.32^c^	1,224.89 ± 793.18^c^
CKMB, (U/L)	11.41 ± 3.53	12.11 ± 2.88	11.22 ± 3.42	131.87 ± 63.45^c^	138.74 ± 57.16^c^

*CAD, coronary artery disease; UA, unstable angina; NSTEMI, on-ST-segment elevation myocardial infarction; STEMI, ST-segment elevation myocardial infarction; SBP, systolic blood pressure; DBP, diastolic blood pressure; PP, pulse pressure; TC, total cholesterol; TG, triglyceride; HDL-C, high-density lipoprotein cholesterol; LDL-C, low-density lipoprotein cholesterol; Apo, Apolipoprotein*.

1 *Mean ± SD determined by t-test*.

2 *Because of not normally distributed, the value of triglyceride was presented as median (interquartile range), the difference between the two groups was determined by the Wilcoxon-Mann-Whitney test. The P-value was defined as the comparison of case and control groups*.

a *P < 0.05*;

b *P < 0.01*;

c *P < 0.001*.

Subsequently, we compared the gene expression levels of these two genes at different times (1, 4–6, 30, and 180 days) after myocardial infarction. We found that in the TLR2 group, the expression of *TLR2* increased significantly in AMI (1 and 4–6 days after MI) but decreased in the convalescent period (30 and 180 days after MI). Similar results were found in the CD14 group, but the expression level of *CD14* in convalescence was still higher than that in the stable coronary heart disease group (Figure 6C).

To further explore these two genes and the occurrence of heart failure after myocardial infarction, we performed a two-factor survival analysis on GSE59867. According to the expression of *TLR2* and *CD14*, the patients were divided into four groups according to the median value of *TLR2* and *CD14*. Survival analysis of heart failure events after myocardial infarction was calculated. As shown in Figure 6D, we found that the incidence of heart failure events was the lowest when the expression levels of these two genes were both decreased, which was statistically significant compared with the other three groups (*P* < 0.01 for all).

**Figure 7:**
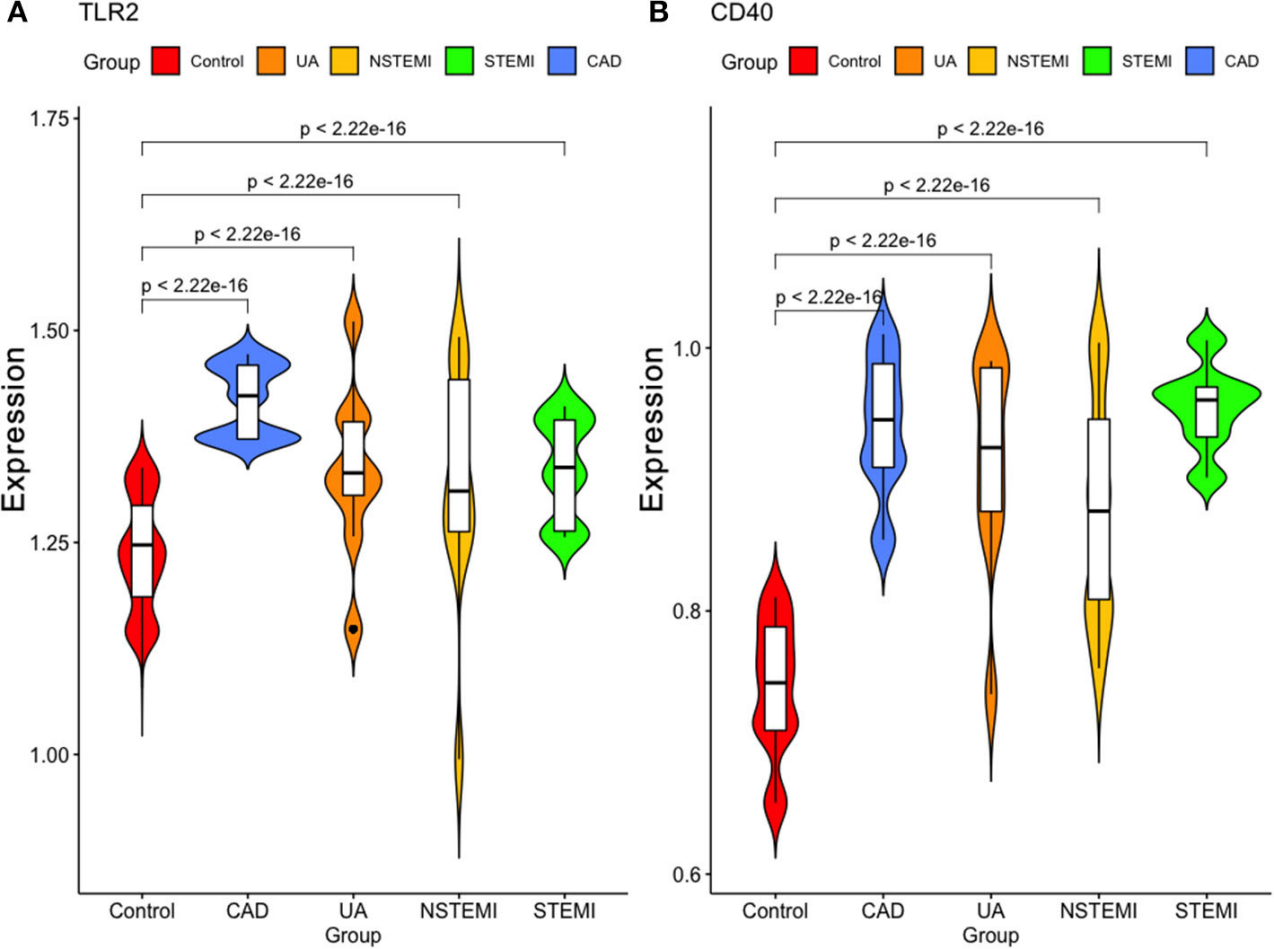
Validation of the expression of these two hub genes in our study sample. **(A)** TLR2 **(B)** CD40.

As shown in Figure 7, we validated these data in a population sample and came to the same conclusion as the previous microarray data. Both expression levels of *TLR2* and *CD14* were lowest in the normal group and were high in the population with coronary artery disease and acute coronary syndrome.

## Discussion

Currently, CAD remains one of the leading causes of death worldwide, and the high morbidity and mortality have become a burden in all countries worldwide, which has gradually attracted people's attention (Benjamin et al., 2018). CAD and gene expression changes are inseparable, and with the continuous progress of technology, the increasing maturity of high-throughput sequencing technology provides a new theoretical basis for our understanding of the pathogenesis of CAD. In the current study, we used integrated WGCNA methods to analyze the dataset and identify two hub genes (*TLR2* and *CD14*) that were positively correlated with the severity of coronary atherosclerosis. Subsequently, we validated these two genes through other datasets and found that they are related to atherosclerotic plaque vulnerability. Increased gene expression is directly related to the onset of AMI and is positively related to heart failure after myocardial infarction.

Toll-like receptors, as a part of the interleukin-1 receptor/toll-like receptor superfamily, are expressed on cell surfaces, where they bind endosomally (*TLR11, TLR12*, and *TLR13*), have microbial membrane components (*TLR1, TLR2, TLR4, TLR5*, and *TLR6*), or recognize microbial nucleic acids (*TLR3, TLR7, TLR8*, and *TLR9*) (Kawai and Akira, 2009). According to previous research, *TLR2* and *TLR4* can be expressed by macrophages, neutrophils, and dendritic cells and activate NF-κB pathways to lead to CAD (Cole et al., 2010). Schoneveld et al. showed that *TLR2* activation increases atherosclerotic plaque formation by using ApoE^−/−^ atherosclerotic mouse molding. In addition, *TLR2* can participate in not only initial intimal lesion formation but also occlusive disease development (Schoneveld et al., 2005). Furthermore, in an IL-6-dependent manner, *TLR2* can promote vascular smooth muscle cell migration from the tunica media to the intima (Lee et al., 2012). Beyond participating in atheroma development, many studies have demonstrated that *TLR2* can activate neutrophils and that free radical production results in coronary endothelial dysfunction after ischemia/reperfusion. This finding suggests that *TLR2* may be associated with new dysfunction after myocardial infarction (Favre et al., 2007; Yu and Feng, 2018). In our current study, we found that *TLR2* is related not only to the severity of coronary atherosclerosis but also to the vulnerability of plaques. *TLR2* is also directly related to heart failure after myocardial infarction. This is consistent with previous research results. This shows that *TLR2* is significantly correlated with the development and severity of CAD.

*CD14*, as an important mediator of inflammatory reactions (Rajasuriar et al., 2015), plays an important role in the process of immunity, inflammation, vascular endothelial dysfunction and atherosclerosis, which have a high risk of contributing to CAD (Hohda et al., 2003). Recent studies on single nucleotide polymorphisms (SNPs) have found that several SNPs of *CD14* are associated with CAD and AMI (Shimada et al., 2000; Raza et al., 2018). Lee et al. found that urinary *CD14* levels were significantly higher in CAD patients than in normal controls, which may be a potential marker of CAD (Lee et al., 2015). However, the specific pathogenesis has not been systematically elucidated. We speculate that inflammation may be induced by *CD14*, resulting in immune dysfunction and endothelial dysfunction leading to atherosclerosis. In our current research, we also found that *CD14* is related to the severity of coronary atherosclerosis and the vulnerability of plaques. In addition, *CD14* could also be directly related to heart failure after myocardial infarction.

First, our data results are only derived from microarray analysis. Although the roles of these two hub genes in the pathogenesis of CAD are described from different dimensions, there is still a lack of further verification. Second, we found that these two hub genes play an important role in the pathogenesis of CAD, but we lack detailed *in vivo* and *in vitro* experiments to verify this hypothesis, which is the next step we need to improve.

In conclusion, 143 sample microarray datasets from the GEO database were systematically analyzed. After finishing integrated weighted gene coexpression network analysis, we found that 560 genes may be associated with CAD or a CAD class. Then, we performed GO, DO functional, KEGG pathway enrichment and protein-protein interaction analyses for these genes, and two genes (*TLR2* and *CD14*) were finally identified. When validated with another microarray dataset and population samples, we found that the alteration in the expression of these two genes is related not only to the severity of CAD but also to acute myocardial infarction caused by the vulnerability of arterial plaque and is also directly related to the occurrence of postinfarction heart failure. The specific mechanism may be related to inflammation and immune dysfunction caused by these two genes, but further large-scale experiments are needed to verify and elaborate the specific mechanism.

## Data Availability Statement

The original contributions presented in the study are included in the Supplementary Material, further inquiries can be directed to the corresponding author.

## Ethics Statement

The studies involving human participants were reviewed and approved by consent was obtained from all subjects for this study, and the Ethics Committee of Liuzhou People's Hospital approved this study (No: Lunshen-2018-KY; Jan. 06, 2018). The ethics committee waived the requirement of written informed consent for participation.

## Author Contributions

BQ and J-HC conceived the study, participated in the design, performed the statistical analyses, and drafted the manuscript. LM conceived the study, participated in the design, and helped to draft the manuscript. C-MZ and G-XD drafted the paper. YW and LT revised the paper. All authors contributed to the article and approved the submitted version.

## Conflict of Interest

The authors declare that the research was conducted in the absence of any commercial or financial relationships that could be construed as a potential conflict of interest.

## Abbreviations

Apo: apolipoprotein
BMI: body mass index
ACS: acute coronary syndrome
CAD: coronary artery disease
CAMs: Cell adhesion molecules
DO: disease ontology
GAPDH: glyceraldehyde-3-phosphate dehydrogenase
GEO: Gene Expression Omnibus
GO: gene ontology
HDL-C: high-density lipoprotein cholesterol
KEGG: Kyoto Encyclopedia of Genes and genomes
LDL-C: low-density lipoprotein cholesterol
MAPK: Mitogen activated protein kinase
MCODE: molecular complex detection
MI: myocardial infarction
PPI: protein-protein interaction
qRT-PCR: quantitative real-time PCR
TC: total cholesterol
TG: triglyceride.
