# Erratum: Integrated weighted gene co-expression network analysis identified that TLR2 and CD14 are related to coronary artery disease

**DOI:** 10.3389/fgene.2023.1347174

**Published:** 2023-12-19

**Authors:** Frontiers Production Office

**Affiliations:** Frontiers Media SA, Lausanne, Switzerland

**Keywords:** gene expression omnibus, integrated weighted gene co-expression network analysis, functional enrichment, functional validation, prognostic analysis, coronary artery disease

In the published article there was an error in the **article title**. Instead of “Integrated Weighted Gene Co-expression Network Analysis Identified That TLR2 and CD40 Are Related to Coronary Artery Disease”, it should be “Integrated Weighted Gene Co-expression Network Analysis Identified That TLR2 and CD14 Are Related to Coronary Artery Disease.”

The publisher apologizes for this mistake. The original version of this article has been updated.

