# Supplementary figures and images for: Integrated Weighted Gene Co-expression Network Analysis Identified That TLR2 and CD14 Are Related to Coronary Artery Disease
Source: Front Genet. 2021 Jan 26;11:613744. doi: 10.3389/fgene.2020.613744 (PMC7870792; doi:10.3389/fgene.2020.613744)

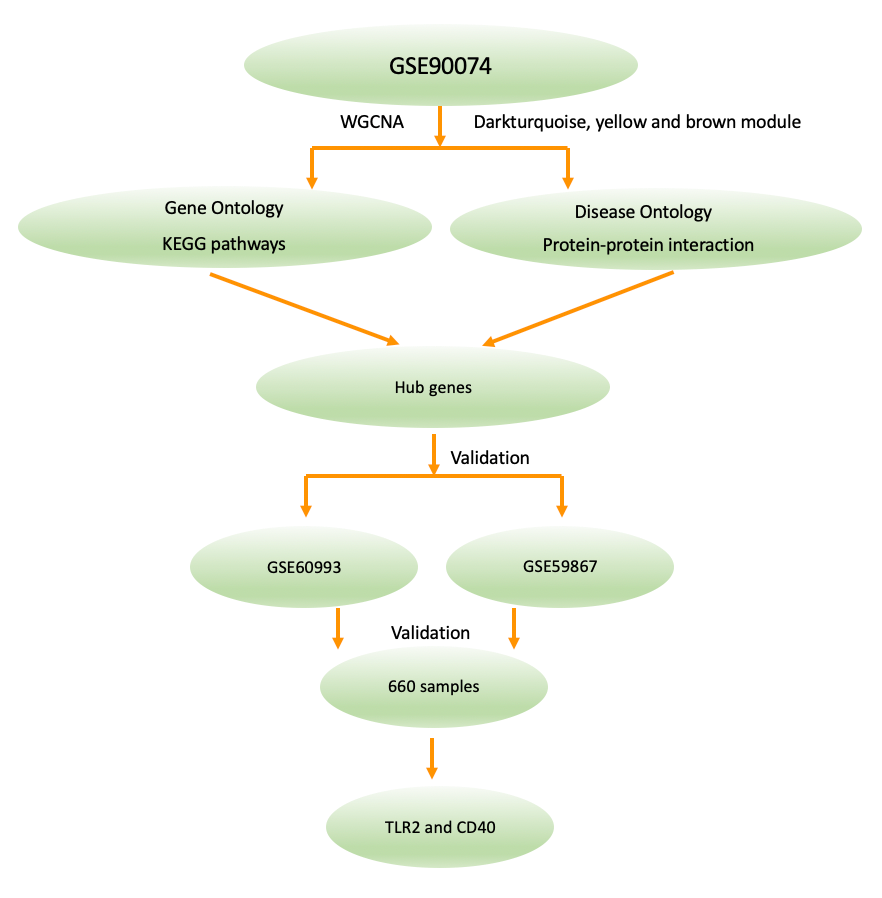

Supplement: Supplementary file 4 [file Image_1.TIFF]

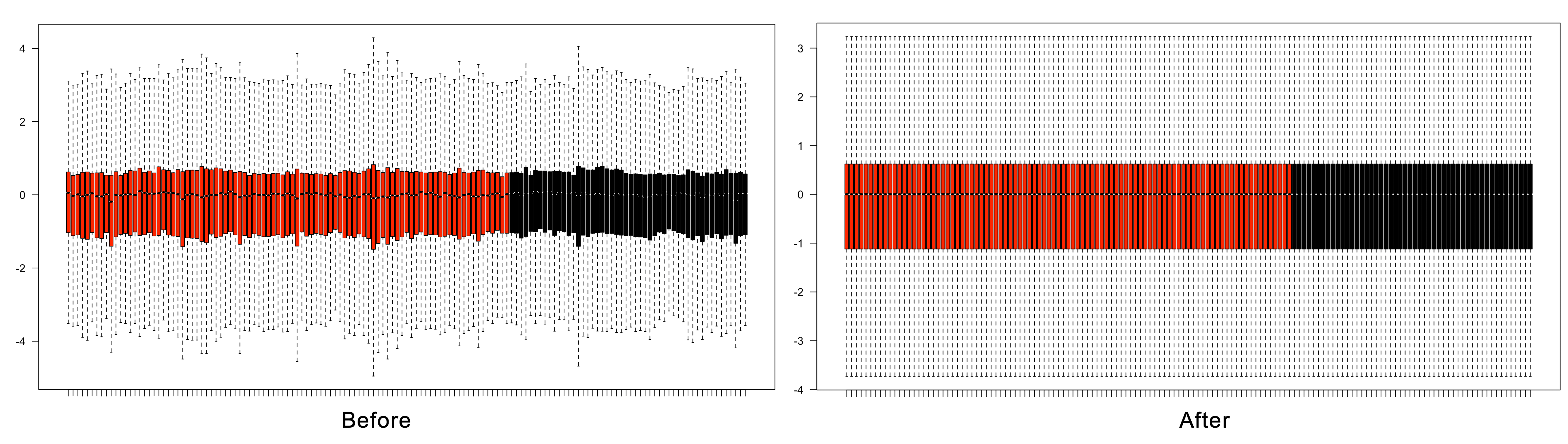

Supplement: Supplementary file 5 [file Image_2.TIFF]
